# Research of cognitive disorders and quality of life in patients, who are receiving methadone replacement maintenance therapy

**DOI:** 10.1192/j.eurpsy.2024.309

**Published:** 2024-08-27

**Authors:** N. Halytska-Pasichnyk, V. Ogorenko

**Affiliations:** ^1^Psychiatric, narcologic and medical psychologic department, Dnipro State Medical University; ^2^Department of replacement maintenance therapy, “Dnipropetrovsk multiprofile clinical hospital for provision of psychiatric care” Dnipropetrovsk regional council”, Dnipro, Ukraine

## Abstract

**Introduction:**

Important goals of substitution therapy include: reducing the desire to use opioids - methadone enters the brain with a minimal euphoric effect, reduce the desire to use opioids, allowing to avoid the risk of overdose and control their addiction; prevention of withdrawal syndrome; improving the quality of life - can contribute to the restoration of patients, allowing them to return to a normal life, improve their social, professional and family situation; reducing the risk of transmission of infections HIV and hepatitis; reducing crime - control addiction can reduce related crime and to illicitly obtain opioids; psychosocial support helps patients develop coping strategies and increases their chances of long-term recovery.

The goal of substitution therapy is not to completely get rid of addiction, but it can help stabilize the patient’s life and facilitate the recovery process.

**Objectives:**

Many patients receiving MT also have mental disorders such as cognitive decline, depression, anxiety, PTSD, or even bipolar disorder. These conditions can greatly affect the course and results of treatment.They may also have problems with employment, housing, family conflicts, and legal issues.

**Methods:**

In the course of the study, 134 patients aged 26 to 64 years (105 men and 29 women) with a diagnosis of opioid addiction and receiving methadone therapy were examined. Of them, 48 patients had a period of stay at MT of up to three years and 86 – more than three years. The Montreal Cognitive Scale (MoCA) was used to assess comorbid cognitive impairments. The WHOQOL-BREF questionnaire was used to assess the quality of life.

**Results:**

The range of indicators of cognitive functions varied from 21 to 29 points (average - 25.3). 61 patients (46%) showed a result of 26 and above, indicating the absence of cognitive impairment, 51 patients (38%) received from 24 to 21, indicating moderate cognitive impairment. 22 patients (16%) had borderline indicators.

When assessing the level of quality of life, indicators of physical and psychological components varied from 12 to 31; self-perception in the range from 10 to 27 points; microsocial support from 3 to 14 points; social well-being from 11 to 36. In general, the level of satisfaction with the quality of life was in the range of 38-83%.

**Image:**

**Figure:**
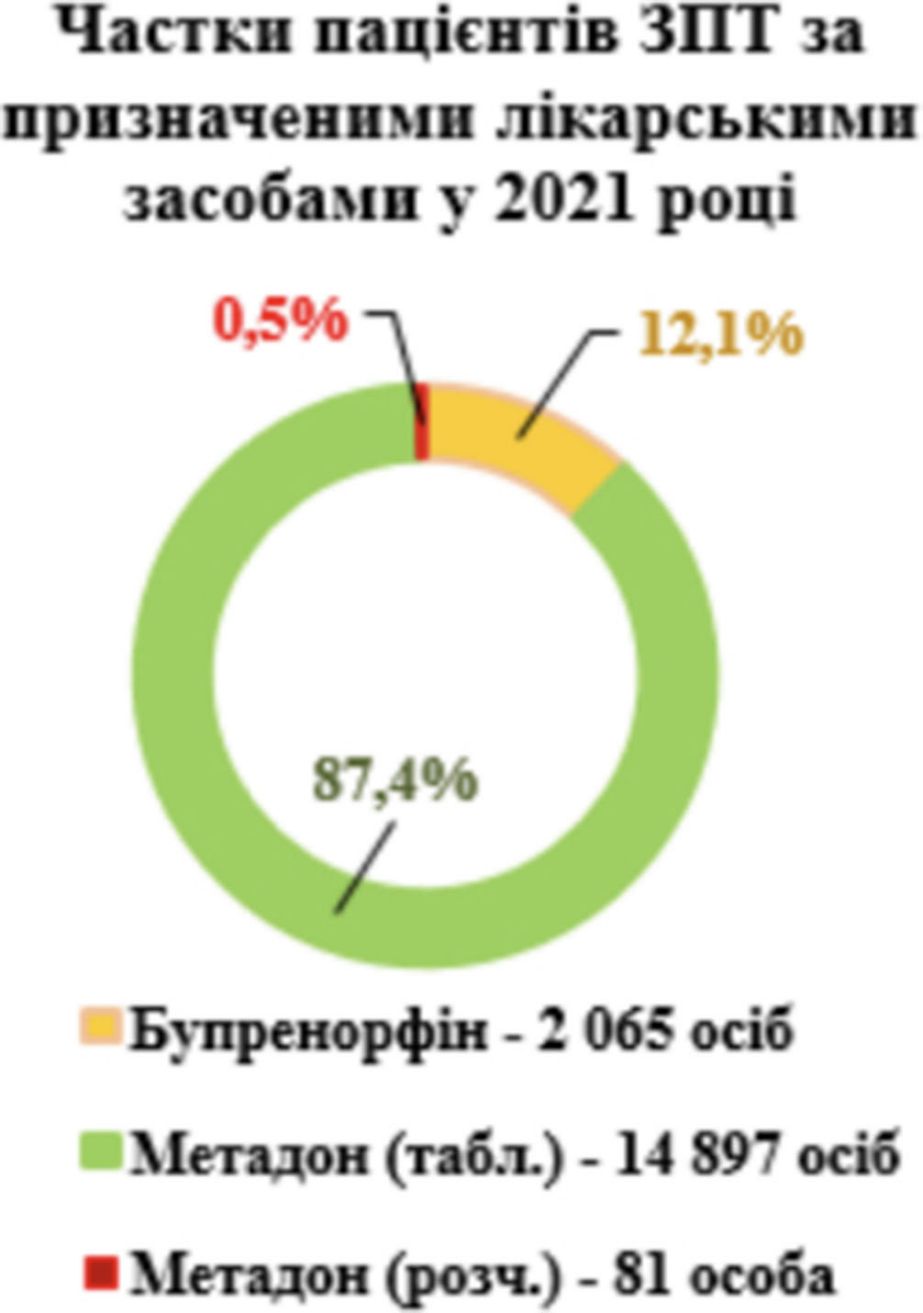

**Image 2:**

**Figure:**
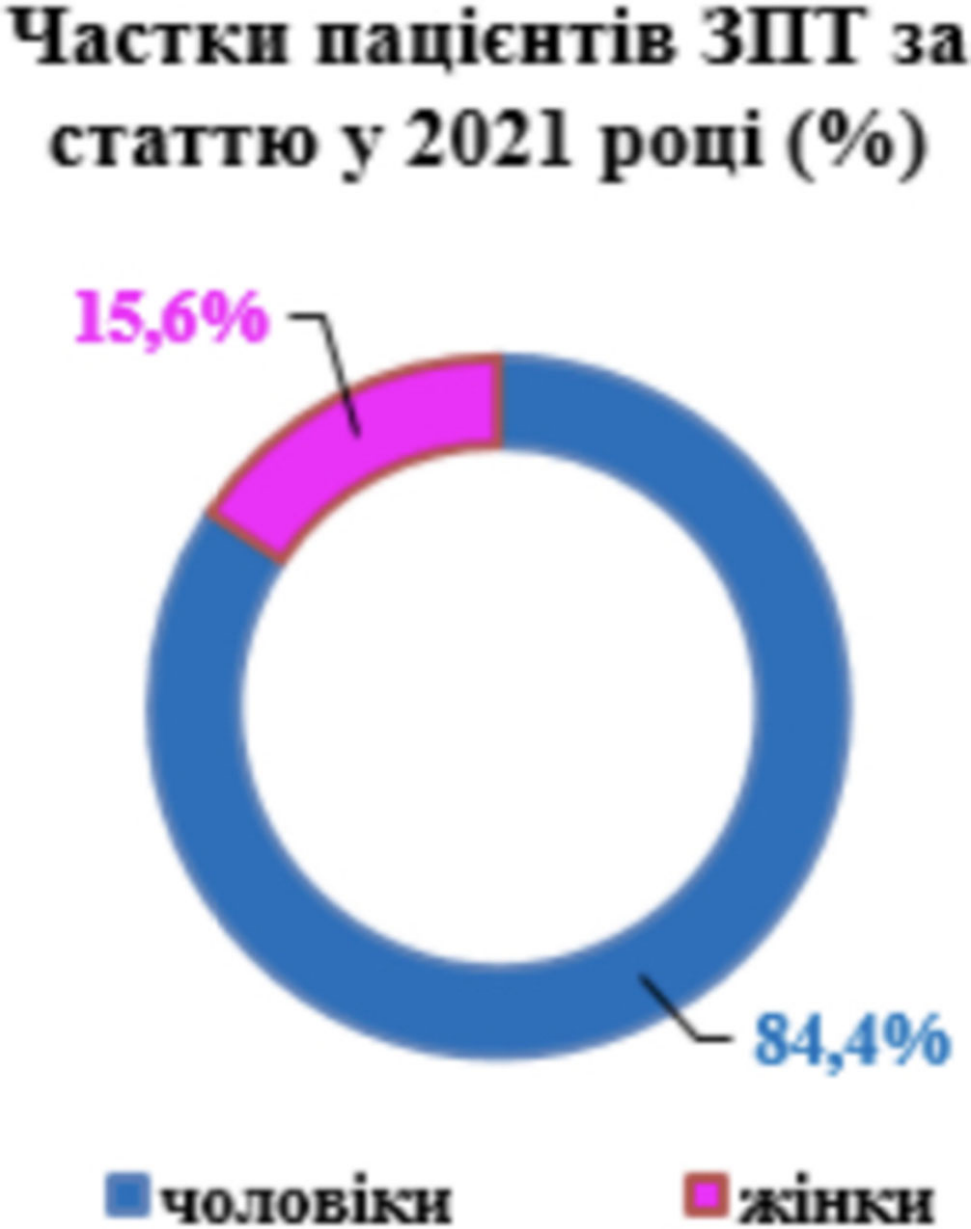

**Conclusions:**

Opioid addiction therapy should be consist of an assessment of physical and psychological status, comorbid disorders, quality of life, etc. We can see, MT does not significantly affect the cognitive functions. The differences in the assessment of the quality of life were noted in the components of microsocial support and social well-being, which indicates the vulnerability of patients in these areas. Duration of opioid dependence, availability of psychosocial support, presence of comorbid conditions affect the quality of life. It is important that treatment is tailored to individual needs of patients.

**Disclosure of Interest:**

None Declared

